# Regret Now, Compensate It Later: The Benefits of Experienced Regret on Future Altruism

**DOI:** 10.3389/fpsyg.2022.840809

**Published:** 2022-04-07

**Authors:** Teng Lu, Dapeng Liang, Mei Hong, Jiayin Sun

**Affiliations:** ^1^School of Management, Harbin Institute of Technology, Harbin, China; ^2^School of Humanities, Social Sciences and Law, Harbin Institute of Technology, Harbin, China

**Keywords:** regret, selfishness, dictator game, emotions, social discounting

## Abstract

This article explores how experienced regret and relief evoked in a risky gambling task influence subsequent intertemporal pro-social behavior. We apply a dictator game experiment with delayed rewards to investigate the effect on donating behavior by simultaneously the time delay when the recipient accepts the donation and the emotions experienced by the participant. We examine this effect using a choice titration procedure. The results reveal that independent of the prior experienced emotions, participants’ donations decrease as the time delay rises; the hyperbolic model provides a better explanation of this finding. Significantly, experienced regret impacts the shape of the social discount function with delayed rewards, which is reflected in notably different discount rates. Participants who experienced regret exhibit a lower discount rate than those in the relief condition. Note that this distinct type of generosity differs significantly at the 14-day delay but not at the shortest and longest. It follows that regret can promote future altruism and intertemporal pro-social behavior, depending on the delay.

## Introduction

Most pro-social behaviors involve intertemporal trade-offs, where people need to weigh costs and benefits at different points in time (Yi et al., 2011; Chopra et al., 2021). In this process, emotions experienced at the moment of decision-making (i.e., immediate emotions) serve as one of the primary forces driving interpersonal behavior (Forgas, 2016; Pérez-Dueñas et al., 2018). The ever-changing emotions experienced in daily lives provide helpful input informing people’s altruistic motives and interpersonal strategies (Barthel et al., 2018; Christner et al., 2020; Zaki, 2020).

This paper focuses on the immediate emotions, namely experienced regret and relief. Regret theory by Bell (1982) states that regret and counterfactual thinking are considered to be exceptionally informative in that people can learn from their faults and rectify their behavior based on their experienced emotions (Pieters and Zeelenberg, 2007; Epstude and Roese, 2008; Kutscher and Feldman, 2019). Counterfactual thinking itself has been shown to benefit in terms of subsequent problem solving and performance enhancement (Epstude and Roese, 2008; Henne et al., 2021). Furthermore, when people learn about the causal relationship between past actions and present outcomes, counterfactual thinking and hence regret may help them put events into context and thus make “sense” out of the past (Pieters and Zeelenberg, 2007; Sim et al., 2020). The experience of regret has been associated with meaningful decisions (Igou et al., 2018; Sijtsema et al., 2021), for instance, risky decisions, overconsumption, and obesity epidemic (Chun et al., 2019; Davvetas et al., 2020). Also, intense and persistent regret may negatively affect the psychological and physical aspects (Fredrickson, 2001; Leger et al., 2020). In summary, regret experiences, due to their functional characteristics, are valued more than other negative emotions (Saffrey et al., 2008), understanding how people experience and manage regret is a research task with real-life implications.

Emotions have essential social functions and regulate social interaction (Martinez and Zeelenberg, 2015). Unfortunately, although previous research has demonstrated the powerful influence of emotions on improving and motivating interpersonal strategies, empirical research on the role of emotions in social decision-making still discloses some limitations. A plethora of evidence has thus far focused primarily on the “value” aspects of emotions (i.e., their positive or negative aspects) and their impact on subsequent decision-making. However, those research abstracts the time signature of pro-social behavior, limiting the explanatory scope of emotions on interpersonal strategies and not being representative of the real world. To better understand the impact of emotions on social discounting in the real economic world, we plan to experimentally investigate how a person’s generosity depends on previously experienced emotions, as well as on the temporal distance between the participant and the recipient. This research is appealing for the following two reasons:

First, the question of how emotions affect interpersonal strategies and selfishness in basic, simple allocation tasks has not been empirically addressed. Research has shown that emotions are one of the primary forces driving interpersonal behavior (Fiedler, 2001; Forgas, 2002), and low-intensity emotions, in particular, can have subtle but lasting effects on decision-making and action (Forgas, 2007; Jin and Oh, 2021). Although many studies have confirmed the functional role of emotions in improving and facilitating interpersonal strategies, findings remain largely inconclusive, suggesting that both positive and negative emotions can promote altruistic and pro-social behavior, depending on the context (Telle and Pfister, 2012; Cavanaugh et al., 2015; Aknin et al., 2018). It was found that people tended to adopt dual-process strategies for decision-making under different emotions (Koch et al., 2013). Dual-process theories suggest that positive emotions promote more assimilative, internally focused processing style, whereas negative emotions promote a more accommodative, externally oriented processing strategy and thus more attention to social norms (Smith and Neumann, 2005). It means that people tend to be more generous in helping others when they are negative emotions. Consistent with the dual-process theories, current research supports that affective states may impact interpersonal strategies by (a) influencing the valence of the responses considered (informational effects) and (b) impacting the strategy of the information processed (processing effects). A recent meta-analysis of positive and negative emotions on selfish preferences suggests that people with positive emotions, when handled properly, are more likely to follow their internal impulses and, therefore, exhibit selfishness in their allocations. In contrast, sad emotions should increase fairness by promoting tolerant thinking and greater attention to external norms (Tan and Forgas, 2010). This view is also consistent with recent research that suggests that people in a negative mood have better memory accuracy (Simpson and Sheldon, 2020), reduce stereotypes (Hall et al., 2019), and are likely to increase fairness and produce a variety of interpersonal benefits (Matovic and Forgas, 2018; Forgas and Matovic, 2020). Based on the above theories (Raeva et al., 2010; Tan and Forgas, 2010; Forgas, 2013), we postulate that experienced regret would increase fairness in the dictator game.

Second, social discounting may be significantly impacted by time delay (Yi et al., 2011). As per Madden et al. (2010), both temporal and social discounting are related to the “extension of the self”: time discounting depends on the extent to which the self extends in time, whereas social discounting depends on the extension of the self in the social domain. A subset of studies provides empirical support for the association between intrapersonal and interpersonal dilemmas (although any implication of such support is speculative, and similarities between intertemporal and interpersonal behaviors are not universal). Temporal discounting as assessed through the intertemporal decision-making process is positively correlated with cooperation in the prisoner’s dilemma game as assessed through the interpersonal decision-making process (Yi et al., 2005; Locey and Rachlin, 2012). Furthermore, it was demonstrated that the regions of the human brain responsible for projecting oneself into the future are associated with the regions that project oneself into the perspective of others (Buckner and Carroll, 2007; Inoue et al., 2021). These arguments are consistent with Construal Level Theory (Trope and Liberman, 2010), which views social distance and temporal distance as dimensions of psychological distance and thus have similar effects on decisions. These instances illustrate that the common practice of modeling pro-social behavior as atemporal hence severely limits the scope of our understanding of pro-social behavior in practice. Given people’s time insensitivity and the negative effects of time on altruism (Ebert and Prelec, 2007; Kovarik, 2009), we predict that participants’ generosity should decrease as time delay rise.

We aim to investigate the impact of prior experienced regret and relief on subsequent intertemporal pro-social decision-making. To our knowledge, this would be the first attempt to interconnect the findings on experienced regret and relief with the domain of intertemporal-interpersonal choice (self-control vs. altruism). The experimental design, referring to previous studies (Jones and Rachlin, 2006; Hayashi and Tahmasbi, 2020), has used a choice titration procedure from psychophysics. Participants are confronted with two types of experienced emotions, regret or relief. First, we hypothesize that generosity would decrease as a delay function independent of experienced regret, and the hyperbolic function should provide the best fit for discounting behavior. Second, we postulate that experienced regret would affect the shape of the social discount function with delayed rewards, which may be reflected in significantly different discount rates.

## About the Experiment

We conducted an experiment combining the experimental paradigm used by Coricelli et al. (2005) with an intertemporal pro-social decision task. We presented participants with a series of trials in which each trial consisted of a risky gambling task (Stage A) and a dictator game experiment with delayed rewards (Stage B). Stage A presented participants with risky gambles in the form of “wheels of fortune” that were equal in probability but differed in the payoff gain or loss size. Based on this setup, the task provided two different types of feedback. In the partial feedback condition, only the outcome of the selected gamble was displayed, whereas, in the complete feedback condition, the outcomes of both gambles were shown. Stage A was adopted from Coricelli et al. (2005). Using this design for emotion manipulation, we ensure that the experience of regret and relief comes from the complete feedback condition: the information given in the counterfactual revelation strongly and systematically moderates the emotional experience regarding the actual outcome, assuring the robustness of the emotion measure. That has been verified in numerous studies (Burnett et al., 2010; Bediou et al., 2011; Corbett et al., 2021).

Stage B was a dictator game with delayed rewards. We tested whether the type of feedback in high-risk gambles impacted the way people traded off between selfish and generous options. We hypothesized that the different emotions elicited by the different complete feedback conditions in Stage A would influence the decision process regarding future altruism in subsequent decisions differently than the partial feedback conditions.

In this paper, we distinguish experienced between decision-related and unrelated experienced regret. Although previous research has confirmed that experienced regret plays a crucial role in subsequent decisions, this influence is mainly reflected in repeated decisions across the same domain. In this paradigm, individuals incorporate previously experienced regret in a decision domain into subsequent decisions when making decisions in the same domain, which is referred to as “decision-related” experienced regret. By contrast, regret is experienced when making the decision, but it is aroused by sources that are objectively unrelated to the decision at hand (cf. Raeva et al., 2010). We call this regret “decision-unrelated” experienced regret, similar to the effects of emotions produced by the music playing in the background of a café, enjoying the breeze on the beach, watching a tragic movie, etc. In parallel, emotions have been shown to persist beyond the induced scenario (Clore and Huntsinger, 2009). According to the appraisal-tendency theory (Lerner and Keltner, 2000), the specific form of the carry-over depends on the underlying appraisal pattern of the particular emotion. Following this theory, we suppose that experienced regret unrelated to the decision, although aroused in Stage A, would be carried over to the next unrelated Stage B.

In light of the underrepresentation of the empirical literature on decision-unrelated regret, we employ a novel approach to examine the consequences of experienced regret in this study and complement this body of evidence. Our task of measuring delayed altruism takes this emotion as a starting point because, within such a paradigm, participants can make informed choices that still allow for the revelation of counterfactual outcomes.

## Materials and Methods

### Participants

We recruited a total of 61 right-handed participants from different faculties of undergraduate, master, and doctoral students (29 female; mean age = 23.30, SD = 4.02). Four participants (two female) were excluded from the initial sample due to a misunderstanding of the experiment. We calculated a prior sample size using G*Power (Faul et al., 2007), considering the Wilcoxon signed-ranks test (within-subject, experienced regret vs. experienced relief) as the main statistical test. The power calculation resulted in a minimum of 47 participants needed to achieve an alpha of 0.05, a standard power $\left(1-\beta \right)$ of 0.90, and an effect size of 0.50. Therefore, we were confident that the experiment had enough valid participants. The study was ethically approved by the Research Ethics Board of Harbin Institute of Technology and conducted following the Declaration of Helsinki guidelines. Participants’ privacy and rights were protected, and informed consent was obtained before the study began.

### Experimental Design and Procedure

Upon arrival, participants (1) were randomly seated in computer cubicles that ensured anonymity, (2) sat in front of a computer screen and received instructions written on paper, and (3) learned a training session consisting of 10 trials identical to the experimental trials. All experimental sessions were conducted on a computer, using the experimental platform jsPsych (de Leeuw and Motz, 2016). The experiment lasted approximately 45 min (participants completed a questionnaire after the test). The identities of participants and recipients were kept anonymous during and after the experiment.

This study used a multifactorial within-subject experimental design to examine the effects of experienced regret and relief on the social discounting of delayed rewards. Each participant was required to participate in 84 trials (2 emotional feedback: regret and relief × 7 temporal distances × 6 monetary amounts of selfish choice). Emotion, temporal distance, and selfish amount were the independent variables. In stage A, the complete feedback was divided into regret feedback and relief feedback. In the complete feedback emotion manipulation, this was evidenced by the difference between the outcome of chosen wheel and the unchosen one: a negative difference defined regret, and a positive difference defined relief. Participants were not told in advance what type of feedback they would receive. In Stage B, the social discounting component employed the procedure initially proposed by Jones and Rachlin (2006), but with the addition of a delay factor. The dependent variable was the average amount of money forgone (to be discussed in detail in Results section).

**Figure 1:**
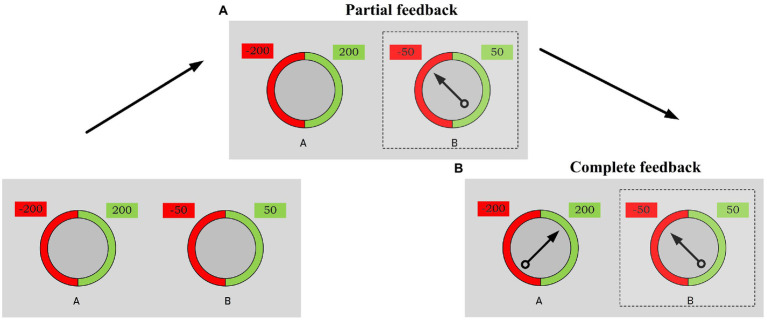
The regret gambling task is similar to the one used by Coricelli et al. (2005). The colored areas on the two wheels represent the probabilities (50/50) associated with monetary gains (green) and losses (red). A dashed box emphasizes the selected wheel. **(A)** Represents a partial feedback condition, where the outcome is provided only for the chosen wheel (the stopping position of the arrow (in black) indicates the result of a loss). **(B)** Represents the complete feedback condition, where the results for both wheels are revealed.

In Stage A, participants were presented with two wheels of fortune (Figure 1). Next, participants were asked to choose one of the wheels to get the maximum score. Both wheels were used throughout the experiment. The position of the wheels on the screen was counterbalanced randomly.

In each gamble, the probabilities associated with winning and losing were represented by the relative colors of the parts of the wheel: gains (green) and losses (red). During the decision process, the probability level remained constant (50/50) for both wheels. That is, the probability of gain or loss through decision-making was the same for partial and complete feedback.

Participants used the mouse to make a choice. Once the participant had selected a wheel, the arrow spun on it and then stopped to show the score of this gamble. Positive or negative numbers indicated possible wins and losses on each wheel next to the wheels. In the partial feedback condition that occurred first, the arrow appeared only in the selected wheel, and participants could not see the results of the unselected wheel. In the subsequent complete feedback condition, the second arrow started rotating in the unselected wheel immediately after the first arrow stopped. Participants were shown the results of both wheels but only obtained the number of points indicated by the arrow on the selected wheel.

After each round of Stage A, participants made a second decision in Stage B. Stage B used the social decision task proposed by Jones and Rachlin (2006) with an initial endowment of 200 cents (value 20 RMB). The novelty of the game was to create a temporal distance between the participant and the anonymous recipient by postponing the payment, which was used to represent the time delay between the time of decision and the time of payment. We performed the temporal distance with seven treatments, 1, 5, 7, 14, 30, 60, and 100. The temporal distance was measured using a ratio scale and converted into a scale consisting of 100 icons. The icons on the scale indicated the time delay for the recipient to receive the donation. For example, the first icon on the left represented that the recipient received the donation with a 1-day delay, i.e., the shortest delay. The icon on the opposite end of the scale (temporal distance 100) represented the longest. Note that the time delay was applied only to the recipient. Participants would immediately receive an amount equal to the total amount minus the donations. That is natural because donations generally come from the donor’s income, and it takes time for the donee to receive the donations.

**Figure 2:**
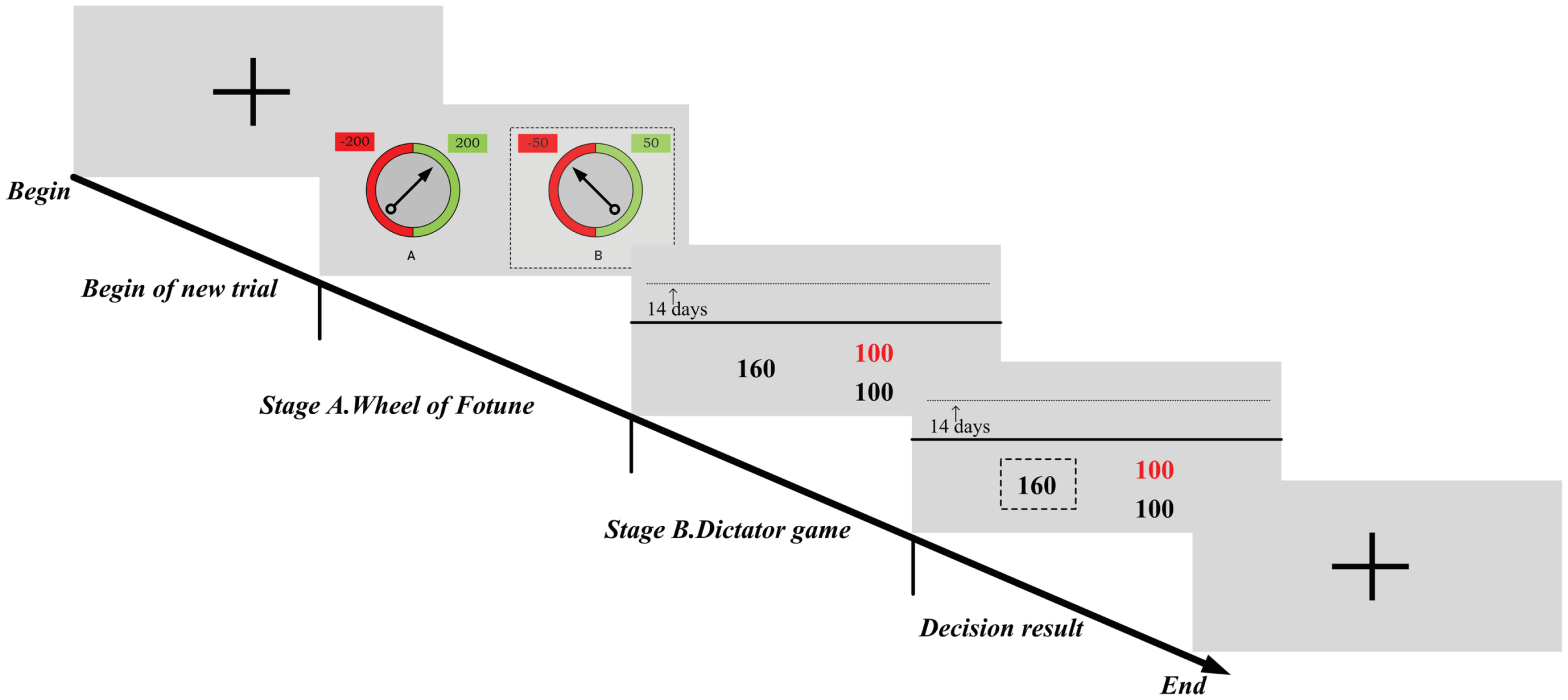
In each trial, there are three screens. The first screen informs about the outcome of the risky gambling task shown in Figure 1. The second screen displays two options, where the option on the left implies a selfish choice and the option on the right implies a generous choice. The black-coded numbers under the scale indicate the participant himself, and the red-coded numbers represent the amount of the recipient’s reward. The third screen presents the decision result.

In each round of Stage B, participants had to choose between selfish and generous choices. The selfish option was a large reward for only the participant, changing in ascending or descending order from 100 to 200 in increments of 20 across trials. The generosity option had a fixed magnitude of 100 and was received separately by the participant and recipient. The arrow on the scale showed the time delay for each trial. The black-coded numbers under the scale in Figure 2 represented the participant and the amount of that reward. The order of each delay treatment was counterbalanced between participants.

The experimental instructions were read aloud in each session, and subjects were given time to ask questions. To increase the salience and relevance of their choices and to make our experiment incentive-compatible, we informed participants at the outset that one of their responses in the dictator game would be randomly chosen and that they would receive the amount they chose plus a five RMB appearance fee. Although the experiment provided a small amount of money, it was real and did not involve deception. Therefore, our experiment meets the criteria for economic research (Schram, 2005).

### Data-Analytic Strategy

First, we calculated the crossover point between selfish and generous choices by titrating the magnitude of the selfish reward. The crossover point was determined by logistic regression, which showed the amount of money the participant was willing to forgo at each delay level. Second, we measured the extent of discounting by the discount rate k . To this end, we used the crossover point to estimate the social discounting of delayed rewards for each participant. Finally, we ran a nonparametric analysis (within-subject Wilcoxon signed-ranks test) between relief and regret condition, aiming to test whether the decision-unrelated experienced relief and regret impacted the participants’ generosity.

### Results

**Figure 3:**
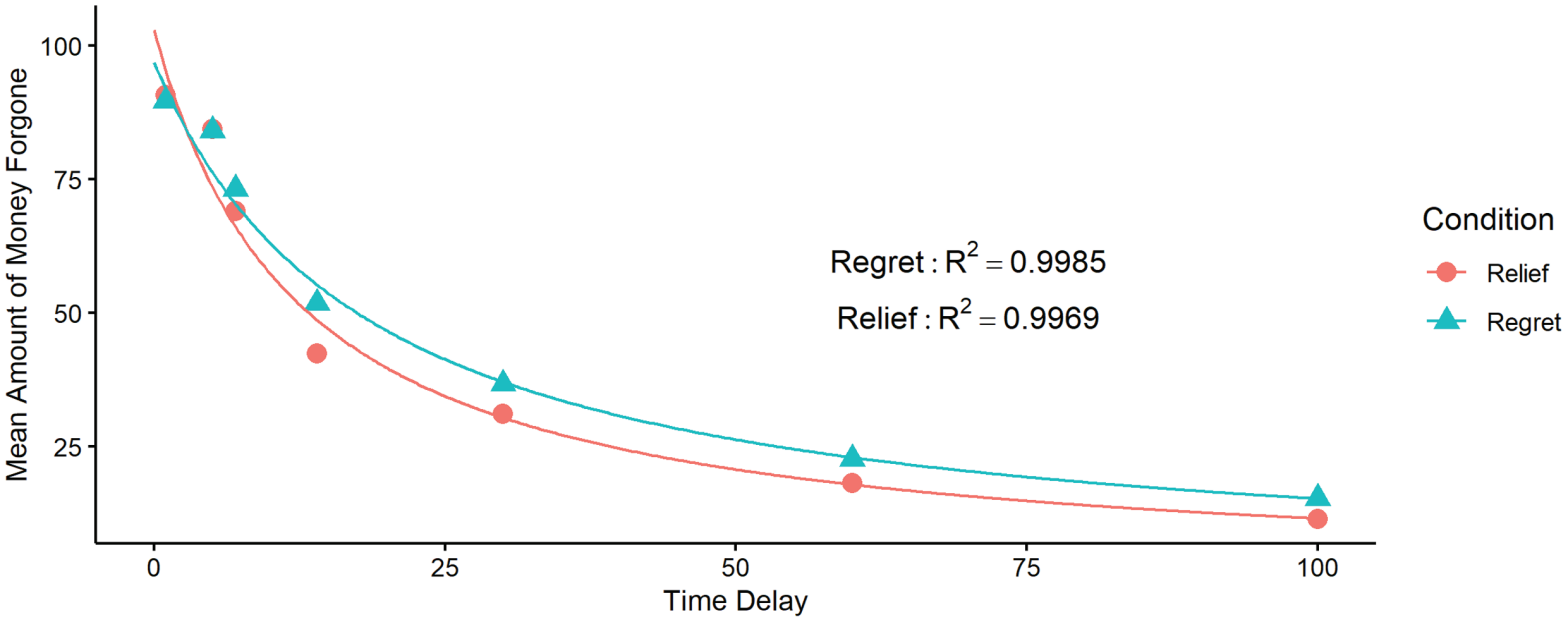
Fitting of the hyperbolic discounting function for the relief (RF) and regret (RT) conditions.

The results of the experiment show that as the time delay increases, participants in each condition are less willing to forgo generous returns, replicating the findings of Kovarik (2009) (see Figure 3). We have used hyperbolic discounting $v_{i}=V/\left(1+kT_{i}\right)$ and exponential discounting $v_{i}=Ve^{-kT_{i}}$ (where $v_{i}$ symbolizes that donations from participant i to the recipient were received after T days) to match the mean value of the forgone amount separately for each condition. The parameters V and k are estimated by nonlinear regression. The results persist that the hyperbolic model has a good fit for two conditions ( $R_{\mathrm{relief}}^{2}=0.9969$ ; $R_{\mathrm{regret}}^{2}=0.9985$ ). Meanwhile, Akaike’s information criterion (AIC) and Bayesian information criterion (BIC) comparisons reflect that the hyperbolic discounting model explains the donating behavior better than the exponential discounting model (as shown in Table 1). Figure 3 depicts the average amount forgone and the hyperbolic fitted curves for each condition.

**Table 1:** The estimation results of RF and RT conditions from the discounting models.

Model	Condition	Goodness of fit	AIC	BIC	Fitted parameters
Hyperbolic model	Relief	*R*^2^ = 0.9969	−65.5946	−65.7569	k=0.07969 V=102.8151
	Regret	*R*^2^ = 0.9985	−75.1449	−75.3072	k=0.05384 V=96.7902
Exponential model	Relief	*R*^2^ = 0.9176	4.1054	3.9431	k=0.02081 V=74.7539
	Regret	*R*^2^ = 0.9358	0.2375	0.07518	k=0.01806 V=78.3948

We find a significant difference in k values across the two conditions ( Z=3.651,p<0.001 ). Participants exhibit a lower discount rate when regret is experienced ( mean:0.06256 ) than when relief is experienced ( mean:0.09705 ). This indicates that, the participants’ generosity levels decay at a slower rate over the time delay in the regret condition.

**Figure 4:**
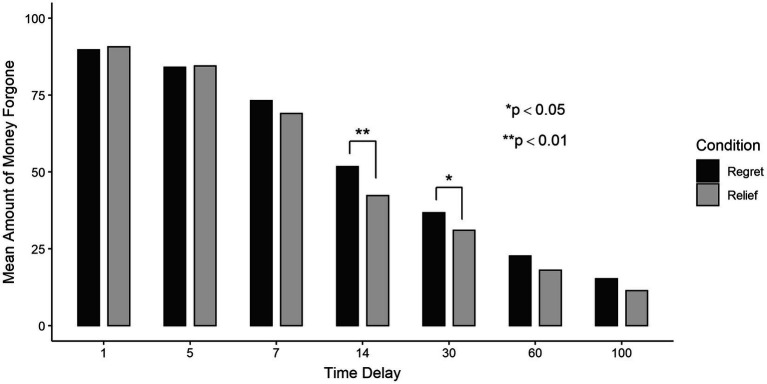
Mean amount of money forgone per delay in the RF and RT conditions.

Furthermore, we also examine the effect of experienced decision-unrelated regret when comparing the level of generosity at each delay. Given the presence of heterogeneous variability, we have conducted several Wilcoxon signed-ranks tests. As summarized in Table 2 and Figure 4, there is a significant difference between the two conditions at the 14-day delay ( Z=−3.018,p<0.01 ). The regret condition ( mean:51.75 ) has a greater mean amount of money forgone compared to the relief condition ( mean:42.28 ).

**Table 2:** The statistical results for each delay between the RF and RT condition.

Delay	*z*	*p*	Effect size	Mean	SD
1	0.472	0.582	*r* = 0.333	RF = 90.70; RT = 89.65	RF = 11.931; RT = 15.807
5	0.147	1.000	*r* = 0.281	RF = 84.39; RT = 84.04	RF = 10.525; RT = 16.460
7	−1.855	0.070	*r* = 0.404	RF = 68.95; RT = 73.16	RF = 9.578; RT = 14.535
14	−3.018	**	*r* = 0.456	RF = 42.28; RT = 51.75	RF = 11.805; RT = 11.514
30	−2.169	*	*r* = 0.509	RF = 31.05; RT = 36.67	RF = 6.991; RT = 15.736
60	−1.896	0.079	*r* = 0.526	RF = 18.07; RT = 22.63	RF = 9.899; RT = 9.733
100	−1.791	0.090	*r* = 0.351	RF = 11.40; RT = 15.26	RF = 6.392; RT = 11.666

* *p* < 0.05;

** *p* < 0.01.

## Discussion

Our research has started with the question of how experienced regret can affect people’s delayed altruism. At first glance, it seemed uncontroversial even if we proposed that experienced regret had a negative impact on altruistic behavior. After all, the assumption of regret as a meaningless “bad” emotion had tacitly guided many past studies (Saffrey et al., 2008). However, we provide empirical evidence that, in contrast to experienced relief, experienced regret is a “good” emotion that promotes fair interpersonal strategies. These facts are consistent with recent research that reports the benefits of negative emotions such as regret on cognitive, motivational, and interpersonal relationships (Forgas, 2013; Matovic et al., 2014; Spachtholz et al., 2014; Lench et al., 2016; Nawijn and Biran, 2019).

The overall results of the experiment support the following findings (Kovarik, 2009; Ogawa and Ida, 2015): the generosity of the participants decreases as the time delay rises. A standard hyperbolic function can describe the discounting behavior in the two emotion conditions. A close analysis of the data reveals that experienced regret appears to affect delay-time-dependent generosity, which is reflected in the fact that participants discounted the rewards of the experienced regret condition at a lower rate. From the perspective of functionalist emotion-cognition theories and dual-process theories (Forgas, 2007; Achtziger et al., 2015), experienced regret may elicit an implicit tendency for participants to reinforce social norms of performance fairness and limit selfish allocation in the dictator game (e.g., choosing the generous option to maintain a reciprocal relationship with the recipient). If so, participants in the experienced regret condition should exhibit a lower discount rate. Our findings support this hypothesis.

Research has demonstrated that time delay is one of the crucial factors influencing future altruism (Yi et al., 2011; Ogawa and Ida, 2015; Andreoni and Serra-Garcia, 2021). By comparing the difference between the two emotions in each delay, we observe a significant difference in generosity at the 14-day delay. Nevertheless, participants’ generosity does not change much at the shortest (1-day) and the longest delay (100-day). It follows that experienced regret is not always beneficial to increased fairness, depending on the delay.

For the shortest delay, participants show an aversion to unfairness and a solid willingness to be altruistic (Osiński et al., 2015). The concepts of time insensitivity and rationality of donations can explain this phenomenon. A considerable body of research has revealed people’s insensitivity to duration (Ebert and Prelec, 2007). An extreme example is a complete insensitivity to the temporal dimension, which corresponds to a zero discount rate over time (Ebert and Prelec, 2007). In the case of the shortest delay, participants may underestimate or even ignore the delay, which makes them give equal weight to their interests and the recipient’s interests. As a result, participants tend to be more generous and behave a type of attitude-behavior consistency. Furthermore, participants may consider the present value of the donations in the near future to be higher than those in the distant future. If so, participants will donate relatively large amounts with only a slight delay. Thus, external emotional factors do not influence participants’ generosity towards the recipient with the closest temporal distance.

For the 14-day delay, the generosity of the two emotion conditions differs significantly. Although regret can improve pro-social behavior, it still needs preconditioning; consequently, the time delay is crucial in determining how experienced regret influences future altruism. According to dual-process theories and processing effects, participants think more assimilatively and may be more willing to follow their internal selfish tendencies in the relief condition. In contrast, when regret is experienced, participants are more concerned with external norms of fairness and tend to make fair allocations. Furthermore, our findings are consistent with prior evidence for the informational effects of emotions (Forgas and Bower, 1987; Gino et al., 2020; Noland, 2021). Several studies suggest that participants who experienced relief tend to recall more positive information and act more optimistically about the current situation. Experienced regret, in turn, by initiating negative information, increases the likelihood of cautious, inclusive, and restrained selfish reactions (Forgas, 2002; Aarhus and Huang, 2020). In the current experiment, participants may have a vivid capacity of episodic recall and a high level of trust with the recipient at the 14-day delay, and involuntary memories led them to generate an intense emotional tenor, thus experiencing regret sparks a more cautious and fair allocation. That may be why participants have forgone more rewards when regret is experienced at the 14-day delay. It implies that external time delays may impact the range for emotion priming and processing effects to occur. So the emotion effect should be strongest when the time delay is 14 days, allowing greater scope for emotion-induced differences in processing style to affect allocations.

Surprisingly, there is no significant difference in generosity between the two conditions at the longest delay. Our interpretation is that the 100-day delay may be a prolonged and uncertain duration for participants, which would trigger an opposite, equally extreme time insensitivity—only the current moment matters, and all future outcomes are assigned zero weight. That is equivalent to the limiting case of an infinite discount rate (Ebert and Prelec, 2007). For the participant, it is wise not to sacrifice an earlier benefit against a delayed cost if they are unsure of a return from others. In addition, long delays are likely to result in participants’ inability to project themselves into the perspective of others (Buckner and Carroll, 2007); hence they have no incentive to maintain such non-reciprocal relationships. In this process, participants exert much more cognitive efforts to overcome the temptation to be selfish than focusing on the experienced emotions (Achtziger et al., 2015). These findings indicate that the experienced regret does not influence participants’ self-focus when the delay is beyond their mental simulations. On the contrary, experienced relief may weaken their focus on external social norms, leading them to freely follow their intrinsic tendency to be selfish (Moore and Loewenstein, 2004; Halali et al., 2013). Thus, participants are selfish with no significant differences in generosity levels at the 100-day delay, regardless of emotion type.

An interesting question in this study is why participants who experienced relief could follow their internal state—ignoring norms and acting selfishly—but participants who experienced regret did not simply follow one norm, namely selfishness or fairness. A more likely explanation is that information provided about the time delay, being undesirable for participants when regret is experienced, fails to invoke an acceptable, alternative common social norm. Therefore, the information behind the prolonged delay may undermine external norms of fairness and restrict regret effects. Instead, information about the short time delay may allow ample scope for regret effects to occur and thus reinforces existing social norms, as described by the experimental results.

Our study has significant practical implications. First, many everyday social situations in our private and working lives involve some conflict between acting selfishly and fairly. Although there is some evidence for the effects of regret on interpersonal behavior in dictator games, the effects of regret on selfishness in a delayed framework have not been addressed previously. The effects of regret on selfishness demonstrated in this paper may have important implications for real-life intimacy, group decision making, and many other everyday situations where experienced regret motivates individuals to approach the task in a constructive way—a subtle interaction between adaptive outcomes of negative emotions and time sensitivity jointly determine their decisions. Second, our study challenges the assumption that positive emotions have universally desirable social consequences. The present study demonstrates that experienced negative emotions also enhance fairness and altruistic sensitivity in interpersonal decision-making, depending on the delay.

A possible limitation of the observations is that the study has measured only one level of social distance, i.e., the recipient is a random stranger. We mainly consider that the dictator and the recipient are strangers about no shared history in the traditional Dictator Game (Hoffman et al., 1994). Meanwhile, it has been found that delay-related self-control and intimacy-related pro-social impulse overlap in a range of decision-making processes, which may cause a blurring of experienced emotion effects (Yi et al., 2011). Another issue concerns the generalizability and the predictive validity of our findings. Although our results are consistent with previous findings and there is reasonable confidence in the reliability of the results, we would prefer to replicate these effects in other studies of social discounting tasks and altruism. Additionally, this paper focuses on the decision consequences of mild emotion states; whether some intense and specific emotions, such as anger, fear, anxiety, and depression, have different effects requires further empirical research (Bishop and Gagne, 2018).

## Conclusion

We summarize the key findings of this study. First, participants who experience regret and relief are willing to forgo a certain amount of money for the benefit of others, and generosity decreases as time delay rises, which is reflected in a hyperbolic function. The analysis further reveals the effect of experienced regret influence the shape of the social discount function. Participants who experience regret exhibit a lower discount rate than those who experience relief. Moreover, this distinct type of generosity depends on the time delay; experienced regret increases participants’ generosity at the 14-day delay, even if altruistic behavior is costly for participants at this point. Finally, our results align with previous work that emotions influence interpersonal strategies and selfishness. Positive emotions may increase self-focus and selfishness; negative emotions may increase concern for others and fairness.

## Data Availability Statement

The raw data supporting the conclusions of this article will be made available by the authors, without undue reservation.

## Ethics Statement

The studies involving human participants were reviewed and approved by Harbin Institute of Technology. The patients/participants provided their written informed consent to participate in this study.

## Author Contributions

TL had the initial idea for the experiment and designed it. TL and MH analyzed the data and wrote the paper. DL contributed to the conception of the work and the interpretation of the data. JS contributed to the conception of the work and the revision of the manuscript. All authors contributed to the article and approved the submitted version.

## Funding

This work is supported by grant 72174043 from the National Natural Science Foundation.

## Conflict of Interest

The authors declare that the research was conducted in the absence of any commercial or financial relationships that could be construed as a potential conflict of interest.

## Publisher’s Note

All claims expressed in this article are solely those of the authors and do not necessarily represent those of their affiliated organizations, or those of the publisher, the editors and the reviewers. Any product that may be evaluated in this article, or claim that may be made by its manufacturer, is not guaranteed or endorsed by the publisher.
